# OLDER ADULTS EXPERIENCING UNSHELTERED HOMELESSNESS IN LARGE URBAN CITY LACK BASIC RESOURCES

**DOI:** 10.1093/geroni/igae098.1043

**Published:** 2024-12-31

**Authors:** Alexis Coulourides Kogan

**Affiliations:** University of Southern California, Los Angeles, California, United States

## Abstract

Older adults constitute the fastest-growing segment of the homeless population in the US. Previous research has found older homeless adults to have more chronic conditions, premature mortality, greater odds of physical disability, and experience accelerated aging compared to housed populations. To inform programs to better serve this population, improved understanding of how they meet their basic needs is warranted. The purpose of this study was to explore the experience of unsheltered homeless older adults with meeting their basic needs for food, drinking water, toilet, hygiene, and healthcare in Los Angeles County. From 2021-2023 (24-months), we conducted a cross-sectional survey using the HOUSED BEDS assessment with people experiencing unsheltered homelessness and initiating care with Street Medicine (N=637). Quantitative data were analyzed to compare adults (18-49 yrs) and older adults (50+ years). Findings show that 40.3% of those sampled were older adults. Most older adults experiencing unsheltered homelessness identified as male (70%), Hispanic/Latino (46.6%), or Black/African American (25.6%). The majority of older adults had health insurance (79%) yet few (22%) had a primary care provider. Older adults lacked 24-hr bathroom access (22.3%), were forced to practice open defecation (82%), and relied on purchased food (50%) and drinking water (40.6%). Maslow’s hierarchy of needs explains that time and energy spent meeting basic needs of survival (including food, hydration, and sanitation) means time and energy not spent elsewhere, such as seeking healthcare. Results of this study suggest a need for scaling Street Medicine which provides basic needs and comprehensive, accessible primary care.

